# Science and Faith to Understand Milk Bioactivity for Infants

**DOI:** 10.3390/nu16111676

**Published:** 2024-05-29

**Authors:** Per T. Sangild

**Affiliations:** 1 Comparative Pediatrics and Nutrition, Department of Veterinary and Animal Sciences, Faculty of Health and Medical Sciences, University of Copenhagen, 1870 Copenhagen, Denmark; pts@sund.ku.dk; 2 Department of Neonatology, Rigshospitalet, 2100 Copenhagen, Denmark; 3 Hans Christian Andersen Children’s Hospital, Odense University Hospital, 5000 Odense, Denmark; 4 Cross-Faculty Center for Science and Faith, Faculty of Theology, University of Copenhagen, 2300 Copenhagen, Denmark

**Keywords:** milk protein, breastfeeding, infant formula, science philosophy, science epistemology

## Abstract

Milk bioactivity refers to the specific health effects of milk components beyond nutrition. The science of milk bioactivity involves the systematic study of these components and their health effects, as verified by empirical data, controlled experiments, and logical arguments. Conversely, ’faith in milk bioactivity’ can be defined as personal opinion, meaning, value, trust, and hope for health effects that are beyond investigation by natural, social, or human sciences. Faith can be strictly secular, but also influenced by spirituality or religion. The aim of this paper is to show that scientific knowledge is frequently supplemented with faith convictions to establish personal and public understanding of milk bioactivity. Mammalian milk is an immensely complex fluid containing myriad proteins, carbohydrates, lipids, and micronutrients with multiple functions across species, genetics, ages, environments, and cultures. Human health includes not only physical health, but also social, mental, and spiritual health, requiring widely different fields of science to prove the relevance, safety, and efficacy of milk interventions. These complex relationships between milk feeding and health outcomes prevent firm conclusions based on science and logic alone. Current beliefs in and understanding of the value of breast milk, colostrum, infant formula, or isolated milk proteins (e.g., immunoglobulins, α-lactalbumin, lactoferrin, and growth factors) show that both science and faith contribute to understand, stimulate, or restrict the use of milk bioactivity. The benefits of breastfeeding for infants are beyond doubt, but the strong beliefs in its health effects rely not only on science, and mechanisms are unclear. Likewise, fear of, or trust in, infant formula may rely on both science and faith. Knowledge from science safeguards individuals and society against ‘milk bioactivity superstition’. Conversely, wisdom from faith-based convictions may protect science from unrealistic ‘milk bioactivity scientism’. Honesty and transparency about the potentials and limitations of both scientific knowledge and faith convictions are important when informing individuals and society about the nutritious and bioactive qualities of milk.

## 1. Introduction and Rationale

**Figure 1:**
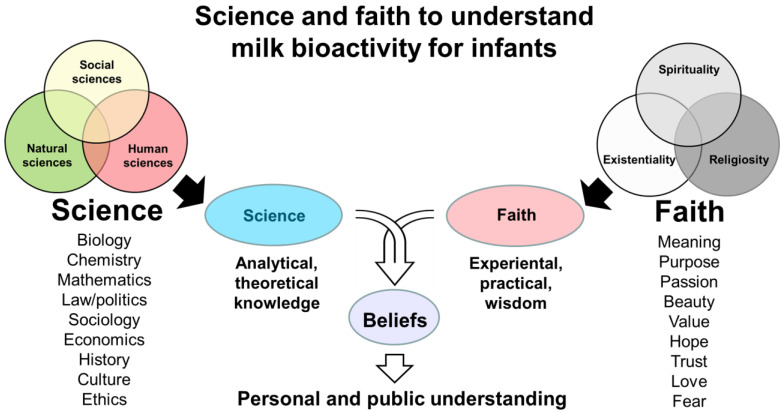
Schematic overview of how both science and faith may form beliefs in milk bioactivity. Here, ‘sciences’ represent all academic disciplines at universities. ‘Faith’ covers secular but also spiritual or religious views and attitudes to human meaning and existence, including health effects of milk (milk bioactivity) for infants. Examples of scientific study fields and faith convictions related to milk bioactivity are listed. Scientific knowledge helps to avoid superstition related to milk bioactivity. Faith elements help to avoid ‘scientism’ related to milk bioactivity, thus preventing unrealistic and exclusive reliance on scientific theories and analyses. Together, science and faith may synergize to form beliefs that determine how to understand and implement milk bioactivity for infant health.

Milk bioactivity can be defined as the effects of the components of milk on human health beyond their role in providing nutrients and energy [1,2,3]. The science of milk bioactivity involves the systematic study of these health effects, as verified via the collection of empirical data and conducting controlled experiments, at least in terms of the natural science of milk bioactivity. Through natural science, we seek to understand the complex physical structures and mechanistic relationships in the natural world. Milk bioactivity is often considered as a research topic in natural science, yet the basic understanding and solutions for society need information from the social and human sciences (humanities). The latter academic domains help to understand milk bioactivity within social, economic, and legal contexts (social sciences) and provide philosophical, aesthetic, historical, or ethical perspectives (human sciences) concerning the use of milk’s bioactives to improve the health of individuals or populations. Hence, social and human sciences are as important as natural sciences to provide ‘scientific evidence’ for milk bioactivity. Similarly, for human health research, knowledge from natural, social as well as human sciences is required to understand and improve human health. Complementarity among sciences is required to understand complex natural phenomena. This paper provides examples of knowledge about milk bioactivity mainly from a natural scientific perspective, yet acknowledging that insights from social and human sciences are equally important. Figure 1 presents an overview of the science and faith concepts presented and discussed in the paper.

In addition to evidence from science, faith-based convictions that are difficult or impossible to verify by science and logic are frequently found among the factors that lead to established beliefs, practical solutions, and health guidelines relating to milk bioactivity. Broadly defined, faith-based convictions include general and secular opinions about nature and milk that are not based on scientific evidence. Faith may also reflect perspectives with spiritual or religious dimensions. Both modern and historical interactions between science and religion are discussed in a large body of academic and popular literature [4]. Using a broad perspective to faith-based convictions, we discuss here the potential and limitations of science in supporting the understanding of milk bioactivity, especially for infants (Figure 1). The paper is part of the Special Issue on “Bioactive Milk Proteins and Human Health” in the journal *Nutrients*. Emphasis is placed on the bioactivity of proteins and peptides, but the health effects of milk may originate from multiple milk constituents and fractions, including carbohydrates, lipids, and micronutrients. Inspiration is gained from the author’s studies on intact milk, milk fractions, or isolated milk proteins in experimental pediatrics [3,5,6,7,8,9,10,11,12,13,14,15,16,17,18,19,20,21,22,23,24,25,26,27,28,29,30,31,32,33,34,35,36,37,38,39,40,41,42,43]. The paper does not provide a review of results from scientific studies, but rather a critical reflection on the scientific methodology and epistemology related to milk bioactivity research.

Medical sciences, including milk bioactivity research, suffer from a “reproducibility crisis”, with vast amounts of work being wasted due to lack of scientific reproducibility. This has led to much speculation on how to improve experimental designs and technology for better reproducibility, but also to broad reflections on the realistic (or unrealistic) attitudes of researchers towards data and the sciences of natural phenomena [44]. Some have advocated for greater intellectual humility in natural science and suggested publication reforms to combat tendencies to oversell results and neglect limitations, uncertainties, and unknowns [45]. Publication and publicity pressures may play a role, but acceptance of the inherent limitations of scientific methods in understanding nature and humans within it is also lacking. This paper argues that knowledge from all three domains of science (natural, social, and human sciences) is required to understand the bioactivity of milk. In addition, faith convictions may complement knowledge from science to deepen the understanding of milk bioactivity effects for human infants across cultures.

First, brief introductions to the concepts of science, faith, belief, and health are given in Section 2, Section 3 and Section 4. Next, some examples of how milk may be viewed as a special food in some world religions are noted (Section 5). After outlining the complex relationship between the numerous bioactive components in milk and multiple health outcomes (Section 6), we discuss examples of the anticipated health effects of milk components, in light of both scientific knowledge and faith convictions (Section 7, Section 8, Section 9 and Section 10). The perspective paper ends with some general reflections on the possible conflicts, complementarities, and/or synergies between scientific knowledge and faith convictions when trying to understand the concept of milk bioactivity (Section 11 and Section 12).

## 2. Academic Sciences Related to Milk Bioactivity

In this paper, we use a continental European definition of science, covering all of the academic disciplines within modern universities (e.g., much more than natural science). Our broad reflections on science in relation to milk bioactivity require a brief introduction to the general characteristics of natural, social, and human sciences at universities. Scientific answers are limited by the methodology within each academic domain of study, and scientific knowledge is not relevant or trustworthy beyond these strict methodological limitations within each field (what is referred to as scientific epistemology). Using scientific methods to investigate the effects of milk protein on human health via the application of natural, social, or human sciences requires awareness, transparency, and honesty about the methodological boundaries of these approaches, especially when seeking applied solutions for individuals and society. Using natural science, the value of identifying biological mechanisms related to new health interventions is often exaggerated, since the requirement for ‘scientific proof’ across all scientific domains fails to be acknowledged. In reality, science cannot prove anything, in accord with the basic falsifiability condition of science proposed by Karl Popper [46]. This presupposition separates actual science from pseudoscience or general public knowledge. Science describes the world in terms of (empirical) observations and human interpretations, following accepted scientific methodology. If hypotheses or assumptions cannot be falsified by empirical data analyses and logical arguments, then we have increased the probability (or belief) that a certain relationship is indeed true. This basic falsifiability condition applies to hypotheses regarding the health effects of whole milk, milk fractions, or specific milk constituents. A principle of both certainty and uncertainty in science [47] is relevant not only for natural sciences, but for science and new knowledge from any academic domain.

**Figure 2:**
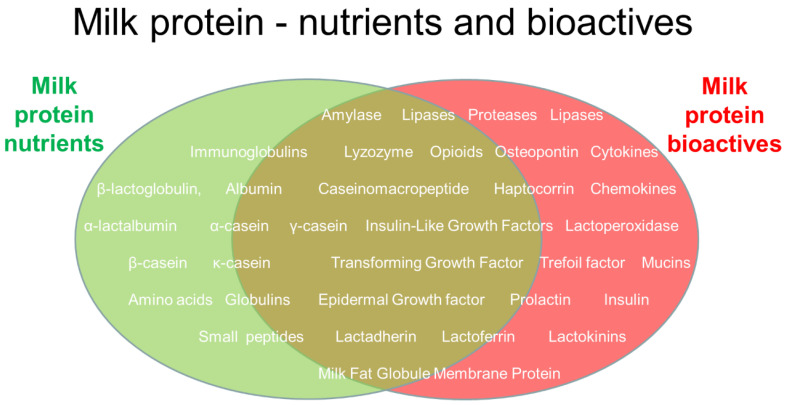
Illustration of some selected milk proteins and their roles as nutrients (indicated by the green ellipse), bioactives (indicated by the red ellipse), or a mix of the two (brown overlapping area). Nutrition effects of milk proteins (e.g., tissue building blocks and energy) interact with roles to regulate body functions and health. Those listed represent only a small fraction of the thousands of known proteins in mammalian milk. Whether a protein is considered a nutrient or a bioactive factor also depends on its digestibility and concentration; both are high for nutrients. In addition to proteins, mammalian milk contains numerous other nutrients and bioactive components, categorized as carbohydrates (e.g., lactose and oligosaccharides), lipids (e.g., glycerides and fatty acids), minerals, vitamins, or other biological categories, interacting with milk proteins and peptides.

From the perspective of natural science, it is known that milk proteins function mainly as nutrients or as health regulators (the latter having no or limited nutritional value in the human body, Figure 2). This paper does not attempt to review all the known structure–function relationships of specific bioactive milk proteins and their complex health effects for groups of humans, including infants. This would be an immense task. Rather, our aim is to discuss how scientific knowledge needs to be complemented by experience from beyond scientific enquiry to reach a conceptualization that can help us to understand how to apply milk bioactivity for infants in multi-cultural societies.

Importantly, the natural science of milk bioactivity aims to understand not only *if* milk proteins affect human health (via observation, data, and health statistics), but also *why and how* milk proteins work (in terms of structure, function, and biological mechanisms). Confirming *both* aspects of milk bioactivity can be very difficult and associated with uncertainties at many levels. In recent decades, a large number of scientific studies have provided evidence (or increased the probability) for the health effects of many specific milk proteins (Figure 2). Many studies have investigated milk fractions rather than single constituents. When the biological efficacy of a single milk protein is demonstrated in natural science, this is often achieved using different types of isolated cells in vitro (e.g., gut, endothelial, or brain cells).

To demonstrate milk bioactivity in infants, fewer studies have been performed using animals or humans. This is explained by the complexity, risks, and economic constraints of conducting in vivo studies. Further, the effects demonstrated in cells in the laboratory are often difficult to repeat at the level of the entire body of animals or humans. Possibly, there are too many health conditions interacting with milk bioactivity in humans, or alternatively, other research tools or insights are needed. In addition, it may be impossible to obtain sufficient amounts of single milk peptides or proteins for investigations through milk fractionation and isolation techniques, potentially requiring difficult safety and efficacy studies of recombinantly derived milk protein products [48]. Finally, when using intact animals or infants, isolated milk proteins or peptides cannot be investigated or tested in isolation from the remaining diet (like in cell studies). Hence, studies include a multitude of interacting effects from other dietary components (milk matrix effects), often leading to ambiguous conclusions. As a result, only a few specific milk fractions or proteins are currently being used to specifically improve infant health, despite the fact that numerous bioactive milk proteins have been identified [49]. This calls for a critical reflection on the natural science of milk bioactivity and its ability to provide new knowledge, as well as its limitations in leading to applied solutions and beliefs relevant to infant health (Figure 1). This article provides such critical reflections to better and more fully understand the concept of milk bioactivity by considering insights from non-natural sciences, as well as from faith convictions.

**Figure 3:**
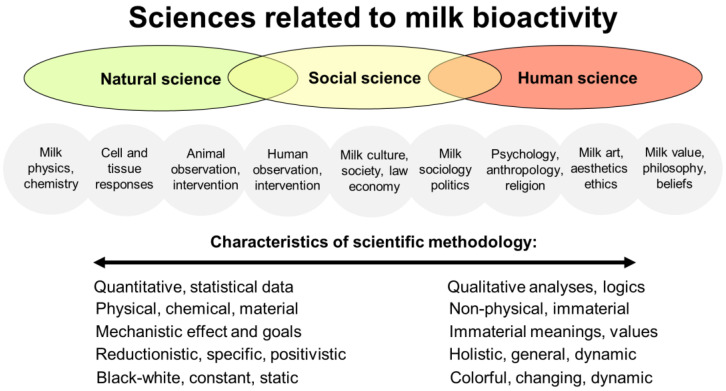
An illustration of the fields of study in milk bioactivity research across natural, social, and human (humanity) sciences. The differently colored ellipses denote the natural sciences, social sciences, and human sciences, as well as their overlaps. The gray circles denote the more specific fields of study relating to milk bioactivity within these different sciences. The text below the circles shows the spectrum of methodologies across the different sciences. Note the overlaps among scientific domains, specific topics, and methodologies, despite their unique characteristics. By research target, and especially by research methodology, social science can be seen as being intermediate between the natural and human sciences. Social science is engaged with studies on both nature and human society and relationships. Social sciences use both qualitative and quantitative research methods. For further information, see Supplementary Figures S1 and S2.

Supplementary Figure S1 lists some general characteristics of the natural, social, and human sciences. Together, perspectives from all three academic domains contribute to a multi-dimensional scientific understanding of milk bioactivity that describes biological, social, and humanistic perspectives—just like other biological phenomena studied at universities. Some research topics contain mixtures of two or even three scientific domains (e.g., human health, psychology, and ecology). Their overlapping natures and main characteristics in relation to milk bioactivity are illustrated in Figure 3. All of the indicated study fields can have milk bioactivity as their prime study target, albeit using very different background knowledge and scientific methodology to reach new results.

The term ‘research’ differs slightly from ‘science’, the latter being based more on well-defined theories and independent, strict methodologies. Historically, this has led classical science to be more detached from the surrounding society than research [50,51]. Research is broader than science and relates more to experience and practical solutions. Science relies on hypotheses and theoretical reflections, with scientific debates (peer-review) being a critical tool in approaching and reaching new scientific truths. Through iterative paths of arguments and counter-arguments, the dialectic process in science leads to new understandings of the world and humans in it. More detailed introductions to contemporary and historical science philosophy in biology and human health are available elsewhere [52]. The remainder of this section outlines some partly overlapping characteristics of the natural, social, and human sciences that relate to milk bioactivity.

Natural science investigating milk bioactivity includes disciplines such as biochemistry, molecular biology, nutrition, and the physiology of body responses. Natural science identifies the exact (often quantitative) physical and chemical composition of milk and its fractions. It characterizes milk bioactive compounds and investigates their mechanisms of action and potential health effects in relation to specific body conditions. In natural science, the focus is on laboratory experiments and mechanisms at the cellular, tissue, organ, or whole-body levels to examine the physiological effects of the components of milk. Natural science aims to uncover structures, define biological mechanisms, and study the interactions between milk bioactive proteins and human health.

Social science focuses on social institutions, their functions, and human social and interpersonal relationships. Social sciences include sociology, anthropology, economics, social geography, political sciences, and public health. Both qualitative and quantitative research methods are used to obtain new knowledge. Social science describes milk bioactivity in the context of human behavior, societies, and cultures. This may include how milk bioactivity is perceived, valued, and utilized in society, as well as studies on the social and cultural factors that influence dairy and milk consumption and assumed health effects. Social scientists study the economic, political, and environmental dimensions of milk and dairy production, distribution, and marketing. Importantly, human intervention and observational studies can be categorized as intermediate between the social and natural sciences (Figure 3), considering the setting-specific design, limited experimental control, many interacting variables, and poor ability to study the mechanistic (cellular) effects of milk.

The topics of human sciences are typically more abstract than those of the social sciences, targeting products and ideas of human culture and behavior with philosophy-based evaluation and interpretation. Human sciences include fields such as literature, language, history, art, music, ethics, religion, theology, and philosophy. Human sciences may seek to understand milk bioactivity from the perspectives of human experience, value, meaning, and ethics, exploring the subjective and existential dimensions of milk consumption and bioactivity. Human sciences examine, evaluate, and interpret milk bioactivity related to its cultural symbolism, rituals, and aesthetic representations with/without ethical reflections. Human science deepens the understanding and logical reflection on the human experience, leading to broader interpretations and implications of milk bioactivity than if conclusions are only based on biology, physiology, or physics/chemistry (natural sciences) or quantitative/qualitative studies at population levels (social sciences). In contrast to the latter descriptive sciences, human science is normative. Human sciences undertake theoretical studies on aspects of human existence that relate to faith characteristics (e.g., meaning, purpose, passion, wonder, values, hopes, trust, love, and fear). Yet, human science remains analytical, theoretical, and highly specific in its research methodology when attaining new knowledge. Human science is a theoretical science based on logic and human reasoning. Human science thereby differs from the practical or experience-based, intuitive, personal, or communal faith-based convictions or ‘common sense’ (Figure 1, right side).

Foundational for all sciences at universities are theory, logic, and reason, resulting in written, peer-reviewed published papers for the advancement of scientific knowledge for individuals and society. Many academic disciplines have sub-specialties that are more concerned with applied problems than logical reflections, thus focusing on practical solutions in society rather than theoretical, written knowledge for peers in science. Such more applied domains (e.g., technology, healthcare, finance, performing arts, and ethics) highlight a natural tension between theory and practice in universities, and between science and research [50,51]. In large research projects, researchers are often encouraged to move across the classical boundaries of theory and practice, and across natural, social, and human sciences in interdisciplinary research efforts, to improve basic understanding, as well as applied solutions. This is highly relevant for science and research into milk bioactivity. Interdisciplinary work helps the awareness of both the potential and limitations of sciences in providing new knowledge about milk bioactivity.

## 3. Beliefs and Faiths Related to Milk Bioactivity

If the combined scientific evidence across natural, social, and human sciences relating to the effects of milk protein on human health is incomplete (which is usually the case), then our basic understanding and clinical decisions to use, or not to use, milk proteins in specific infant conditions will rely partly on ‘belief’— or even ‘faith’. There are no universally accepted definitions of the terms belief and faith, and the words are used interchangeably in the English language. Yet, the two terms differ according to accepted English semantics [53,54,55]. These faith-belief differences provide an important perspective to how science and faith may lead to beliefs that facilitate personal and public understanding of milk bioactivity (Figure 1). Like English, Chinese has two terms that cover belief and faith (信念, xìnniàn, and 信仰, xìnyǎng, respectively). Other languages may have only one term covering both belief and faith (e.g., German: ‘glaube’ and Danish: ‘tro’). However, belief and faith have distinct characteristics and connotations, with different philosophical and applied implications. These differences are important when individuals and the public need to rely on ‘evidence’ and ‘understandings’ of milk bioactivity that are not based on scientific results and knowledge alone.

Belief can be understood as a cognitive, rational state where a proposition is considered as true or accurate, even if that proposition has not yet been investigated or cannot be adequately investigated by science. It involves having confidence in the existence or validity of something, at least temporarily, until obtaining further evidence. Beliefs are required for generating hypotheses in science and may include the belief that science will provide complete answers to all questions in the future [56,57]. For similar reasons, beliefs are important in religions. Beliefs may be based upon personal experience and common sense, but also on scriptural insights, cultural influence, and logical reasoning. Belief can be considered as a form of ‘rationality’ and ‘knowledge’, even while awaiting scientific or other logical proof for specific beliefs. Strong causal thinking in science generates hypotheses about underlying mechanisms based on beliefs, unlike ‘superstition’ [58]. Beliefs are human attempts to provide theoretical, general explanations and ideas about the surrounding world (including milk bioactivity), making beliefs important in both science and religion.

‘Faith’, on the other hand, relies on highly personal convictions about natural phenomena or values that are not possible to investigate or prove by science. Relative to belief, faith is based more on personal experience, intuition, feelings, trust, and hope than on data, observations, and rational arguments [53,54,55]. Faith is not subject to scientific proof or falsification, unlike beliefs. Practical, faith-based expressions can be researched, but faith(s) can never be scientific, according to the normal definitions of science (see earlier). The claim that milk proteins are intimately fine-tuned in their composition by biological evolution and/or divine intervention to fit the needs of suckling infants is a faith, not a science, because such claims are impossible to verify using scientific methods or accepted academic logic. Both belief and faith may include the term ‘common sense’ (‘or reasonable faith’), but faith goes beyond common sense. Faith can be religious or non-religious in nature and based on personal or communal values, affection, hope, and passion. Strong beliefs in the power of science may be denoted as a ‘faith in science’ [56]. On the more negative side, fear can be a strong driver of faith, either fear for certain past/present/future life circumstances or more fundamental fear related to human life, meaning and existence. Faith reflects deep personal and emotional dimensions that involve trust or fear that transcend the observable world. Faith may or may not include spiritual or religious dimensions (Figure 1, Supplementary Figure S2).

Faith in God or gods (religion) can be supported or rejected by rational, cosmological arguments by scholars in human sciences [59]. Yet, faith itself goes beyond rationality and reason. Such a perspective on faith resonates well with an existentialist philosopher like Søren Kierkegaard (1813–1855). To become one self and exist more fully as a person, it is necessary for individuals to take a ‘leap of faith’ rather than rely on reason alone [60,61]. Thereby, a person may need to embrace illogical (although not irrational) prepositions about human existence to become authentic and avoid living in despair [62,63,64]. Kierkegaard emphasized individual, existential choices in life rather than searches for universal truths. This involves an individual act of trust in the face of uncertainty to embrace the unknown. In accord with the psychiatrist Viktor Frankl [65], the individual human quest for ultimate meaning provides a strong ‘faith in life’ or ‘existentiality’ (Figure 1) that goes beyond reason, knowledge, and science in its approach to human life and living.

The importance of not letting faith convictions be in opposition to reason and science is emphasized by many other philosophers. K.E. Løgstrup (1905–1981) argued that everyday human life provides by itself (e.g., not by human free will or choice) ‘sovereign expressions of life’ (e.g., love, compassion, trust, hope, meaning, and beauty) that are closely integrated with, not separate from, human reason and logic [66]. Such complementary or even synergistic views seek to avoid unnecessary dichotomies and dualities between faith and reason. Infiltrated, holistic attitudes to nature’s complexity and its constant and close interaction with human life emphasize respect for experience, beauty, and the meaning of milk for mothers and infants, beyond what is possible to understand by chemical analyses (natural science), social factors (social science), and reflections on human values (human science). The concepts of faith, belief, and reason have been extensively discussed in contemporary and historical science philosophy and theology (e.g., Paul Tillich, Jürgen Habermas, Friedrich Schleiermacher, Georg Wilhelm Friedrich Hegel, Immanuel Kant, and René Descartes [67,68,69,70,71,72,73]). In the present context, faith is used as a broad term reflecting trust, hope, and experience that lead to wisdom that differ from, yet synergize with, knowledge from science in attempts to understand the health effects of milk for infants.

Doubt is fundamental for science, faith, and belief alike. At times, faith may appear resistant to criticism from science, because faith-based convictions do not rely primarily on observable phenomena or logical arguments. Conversely, science may be resistant to inspiration from faith-based convictions, because science relies mainly on theory, observation, and logical reflections to explain the world and humans in it. Unjustified confidence in scientific results and reflections applies not only to natural science, but also to social and human sciences (Supplementary Figure S2). Science and faith are complementary paths to understand the world, not destinations or ultimate goals in their own right. Understanding bioactive milk proteins in human health, including infant feeding practices, may be based on scientific results as well as faith convictions, together forming dynamic and modifiable beliefs relevant to practical solutions (Figure 1). This paper highlights the need to seek this broad understanding by drawing on different fields of science, as well as faith-based existential, spiritual, or religious attitudes to milk bioactivity.

## 4. Physical, Social, Mental, and Spiritual Dimensions of Human Health

**Figure 4:**
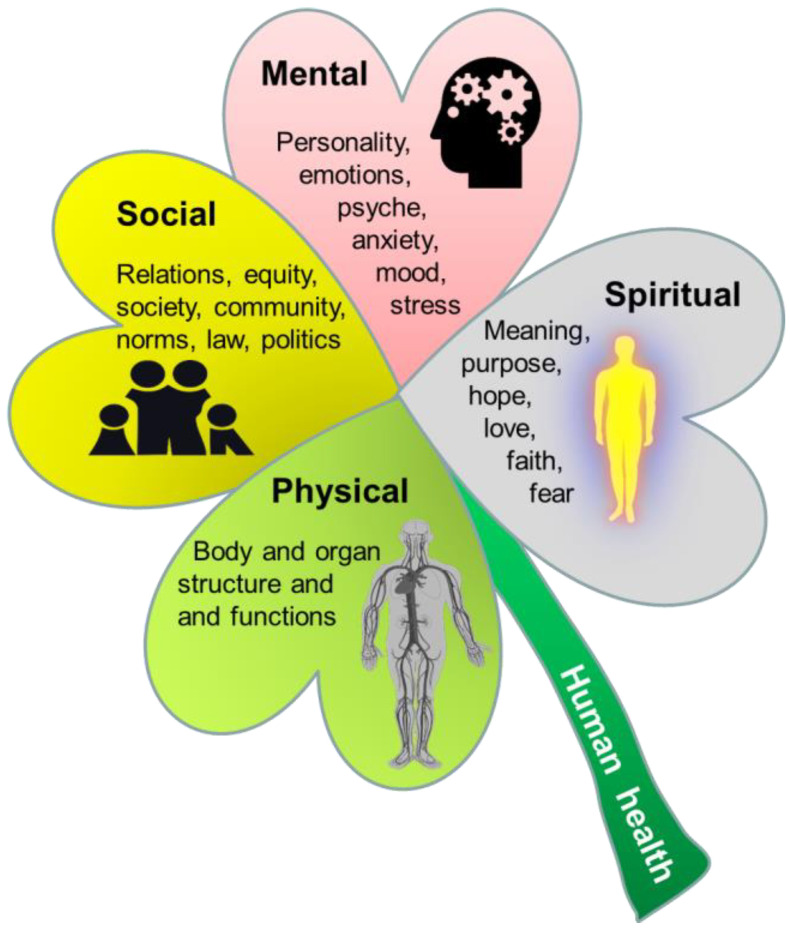
Illustration of the three domains of human health (physical, social, and mental health shown by green, yellow and red ‘leaves’), as defined by the World Health Organization (WHO) [74]. A fourth dimension, spiritual health, has been suggested to be an important addition to the 3-fold WHO health definition [77,78] and is added to the illustration (the grey ‘leaf’). Spiritual health reflects aspects of personal existence, meaning, hope, love, and trust in something greater than oneself, with or without involvement of religious faith. Embedded pictures obtained from Pixibay.com.

According to the World Health Organization (WHO), human health is ‘a state of complete physical, mental and social well-being, not only absence of disease’ [74]. This definition partly reflects the natural, social, and human sciences of health (physical, social, and mental, respectively). Health in relation to society and interpersonal relationships is a well-established research field in medicine (e.g., medical sociology). In addition, attention to human health sciences (medical humanities) is rising worldwide, acknowledging that human health is more than physical and social health [75]. Medical humanities now cover many research fields, not only medical ethics [76]. Figure 4 shows how health outcomes can be divided into four overlapping and interconnected domains of human health.

In addition to physical, social, and mental health, spiritual health has been suggested as a necessary addition to the ‘WHO health triad’ [78]. The concept of spiritual health has a strong tradition in nursing fields and palliative care medicine, but has recently received greater focus in healthcare across the globe [79,80,81,82,83]. Spiritual health can be defined as ‘a state of being where an individual is able to deal with daily events and challenges in a manner that leads to the realization of one’s full potential, including meaning and purpose of life and fulfilment from within’ [78,84]. Within Western medicine, spirituality has been defined by the European and North American Societies of Palliative Care Medicine as ‘the dynamic dimension of human life that relates to the way persons (individual or community) experience, express and/or seek meaning, purpose and transcendence, and the way they connect to the moment, to self, to others, to nature, to the significant, and/or the sacred’ [77]. Spirituality is broader than and differs from religion, which may be defined as an ‘institutionalized pattern of values, beliefs, symbols, behaviors, and experiences that are oriented toward spiritual concerns, shared by a community, and transmitted over time in traditions’ [85]. The concept of spirituality reflects some concepts in Eastern medical systems, most notably the ‘shen’ concept in Traditional Chinese Medicine (TCM). Here, five manifestations of shén (五神) are seen as the basis for all human activities and the source from which the human spirit emerges [86]. Shén embodies the understanding that the physical body, mental activities, emotional life, and spiritual expressions are integrated facets of human health [87]. The long history of the ‘bioactivity’ of herbal remedies and other TCM therapies relies partly on this understanding of spirituality in nature. Until now, shén and spiritual health concepts have been poorly described in the scientific literature, because they fit poorly with modern (Western) models of providing new scientific knowledge and evidence by uncovering structure–function relationships via objective, theoretical, and reductionist human logic and reflections.

Spirituality may or may not be connected with religious (supernatural) faith or practice. As such, it represents a broad, personal, and subjective understanding of the deeper aspects of human experience and existence with or without the involvement of religion [79,80,82,88,89,90]. In this regard, faith and spirituality are closely connected. Both are subject to description by various social and human sciences, yet their very nature and substance are difficult to describe by logic and science. Public health studies (social sciences) can conduct surveys on lived spirituality, but the exact content, as determined by material elements (e.g., brain effects) or its immaterial, personal characteristics (e.g., faith, value, hope, and meaning), makes it difficult for classical natural, social, or human sciences to formulate clear descriptions. This does not exclude the fact that faith, spirituality, and religiosity are real human experiences and profoundly influence how humans perceive and understand milk bioactivity beyond what science can describe and inform. Science provides tools to investigate physical, social, and mental health. Understanding spiritual health requires attention to faith-based views and opinions in addition to scientific descriptions. Faith perspectives can be strictly secular, but also inspired by spirituality or religion (Figure 1 and Figure 4). Before describing the science–faith perspectives of milk protein bioactivity in relation to infants, it is relevant to mention some perspectives on milk bioactivity from world religions. Religious perspectives may, to variable degrees, affect personal opinions, culture and societal traditions for both scientists and the general public.

## 5. Milk Bioactivity in Religions

As will be demonstrated in this section, milk, as the first critical food for all mammals, is, in many human cultures, seen to have special properties beyond science and nutrition. This reflects faith-based (spiritual or religious) approaches to milk, where milk symbolizes purity, nurture, or sustenance. From such faith perspectives, the presence of bioactive compounds in milk can be seen as a manifestation of nature’s complexity or even divine design. When considering milk’s health effects, individuals with a spiritual view towards milk may see bioactive compounds as a representation of the intricate and protected nature of mammalian life and a symbol of the interconnectedness of mother and child.

In Hinduism, cow’s milk is considered as a sacred fluid that can be used in religious rituals and ceremonies [91]. Milk is associated with purity, fertility, and divine blessings. Milk is offered to deities and used in the preparation of sacred substances like ghee (clarified butter) for religious rituals. In the traditional Indian medical system of Ayurveda, bovine milk is considered as a vital ingredient in various medicinal preparations. It is believed to have rejuvenating properties and is used to balance ‘Doshas’ (energies) in the body. Ayurvedic texts describe milk as a nourishing substance that promotes both physical and spiritual well-being: ‘*May four oceans, full of milk, constantly abide in both your breasts, you blessed one, for the increase of the strength of the child! Drinking of the milk, whose sap is the sap of immortal life divine, may your baby gain long life, as do the gods by feeding on the beverage of immortality*!” (Hindu Susruta, III, 10). In Indian Hindu communities, this influences the public perception of milk bioactivity, in addition to results from science, both regarding the view of individual milk bioactive proteins and the perceived relevance of using these alone, as part of milk fractions, or as whole milk, across species (e.g., cows and humans). Importantly, the historical and religious selection of cows as sacred animals in Hinduism is not related to any specific physical, social, or mental health quality of bovine milk. Other animals are also considered to be sacred (e.g., monkeys, elephants, and dogs), despite the fact that their milk, bodies, or activities clearly have minimal nutritional or other benefits for humans [92]. Like Hinduism, many Eastern religions (Buddhism, Taoism, and Shintoism) discourage killing animals for food (especially for monks), but do not discourage drinking milk products from live animals [93]. However, the symbolic and religious meanings of milk appear most explicitly in Hinduism.

In Islam, milk is associated with purity, blessings, and sustenance when mentioned in the Quran and Hadiths: ‘*The mothers shall give suck to their offspring for two whole years* (Quran, Baqara, 2:232; Surah Al-Nahl 16, 66). Islam has particularly strong views concerning ‘milk kinship’ between the mother and her nursing child. Essentially, breastfeeding is seen as the postnatal extension of the fetal period of pregnancy, not only in terms of providing nutrients, social bonding, and mental health, but also relating to the spiritual connectedness between mother and child [94]. Hence, for ‘wet nurses’, it is emphasized that these should be close relatives of the family and that ‘milk siblings’ (genetically unrelated infants nursed by the same mother) should not marry. In Islamic culture, this poses a challenge in terms of establishing donor milk banks, because donor milk is typically derived from many (unknown) mothers [94,95]. Such views clearly inhibit the use of milk bioactive proteins and factions across mothers and from another mammalian species. However, it remains that strong convictions about milk kinship in Islam and some other religions are based on faith, not on scientific knowledge.

In Christianity and Judaism, some scriptural texts can be interpreted as assigning special properties to milk beyond nutrition alone: ‘*Like newborn infants, long for the pure spiritual milk, that by it you may grow up into salvation*’ (1st Peter 2,2); ‘*As he said these things, a woman in the crowd raised her voice and said to him, blessed is the womb that bore you, and the breasts at which you nursed* (Luke 11,27); ‘*Because of your father’s God who helps you, because of the almighty who blesses you, with blessings of heaven above, blessings of the deep that lies below, blessings of the breast and the womb*’ (Genesis 49,25); ‘*Drink deeply of her glory even as an infant drinks at its mother’s comforting breast*’ (Isaiah 66,11). In Christian iconography, the Virgin Mary is often depicted as nursing the infant Jesus, symbolizing her role as a spiritual nourisher (see Section 10) and indicating milk as a connection point between the natural and spiritual worlds. God was incarnated into the human person of Jesus, but from a wider naturalistic perspective, also into nature itself in the form of maternal milk as a ‘sovereign expression of life’ (see Section 3), leading to close entanglement between the natural and spiritual aspects of breastfeeding. Spiritual or religious faith elements play roles in the health views of milk across cultures, even when milk is used symbolically as a metaphor for spiritual or religious dogmas. Such faith attitudes may supplement, complement, or counteract evidence from natural, social, or human sciences (see later).

Milk kinship concerns, e.g., the unwanted effects of milk bioactivity beyond the natural milk transfer from mother to offspring, are based on a combination of spiritual–religious faith, culture, and history, but natural scientific explanations have been suggested. It has been proposed that breast milk transmits genetic material in the form of microRNAs (miRNAs) and stem cells that induce lasting effects on infant tissues at multiple levels via epigenetic regulation [96,97]. This deserves further exploration. However, at present, there is no scientific evidence supporting the belief that mother–infant dyads influence genetic constitution or gene expression via breast milk constituents. First, the number of different miRNAs in breast milk is enormous and they vary widely among individuals and lactation stages. They may or may not be species-specific [98]. Second, it is questionable whether miRNAs are stable (especially after the heat treatment of donor milk) and can be adequately absorbed into the bloodstream. Third, the mechanisms of how miRNAs may affect epigenetic regulation and disease susceptibility are unknown. All of these reservations are also true for bioactivity mediated via intact milk proteins. However, theories on milk miRNA raise important unresolved questions as to whether breast milk can have lasting ‘genetic’ bioactivity on recipients across species. Such theories may increase resistance to the use of cow’s milk products, especially for vulnerable (preterm) infants [99], but also other groups [97,100]. Yet, from a scientific perspective, it remains unknown if milk bioactivity is mother- and species-specific. Beliefs in the health benefits of cow’s milk can be based on scientific evidence [101] and/or religious views (especially in Hinduism). Likewise, fear of dairy products can be based on scientific evidence, as well as faith convictions beyond scientific evidence [97,100,102,103]). At present, the global public understanding of dairy products for human health relies on both scientific evidence and faith-based convictions.

## 6. Milk Protein Bioactivity and Human Health Interactions

**Figure 5:**
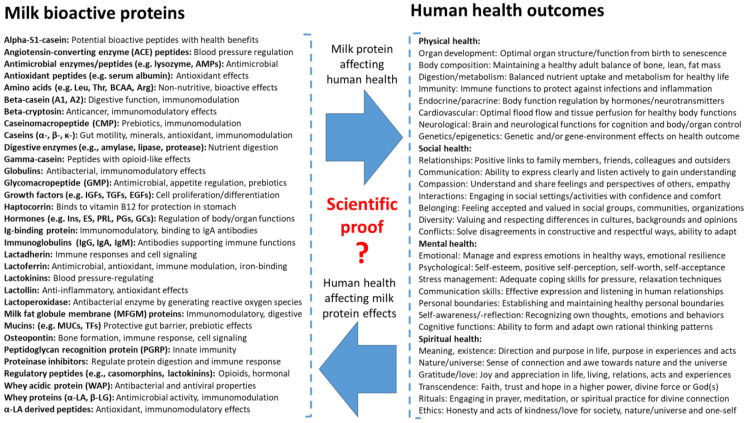
Overview of some milk bioactive proteins (left side) that may affect human health (right side). The blue arrows denote the relationship between these two aspects, influencing each other. The protein list is not complete and may include many others [48,49,107], varying among species, stages of lactation, and health states. When health outcomes include not only physical health (investigated by natural science), but also social, mental, and spiritual health (Figure 4), the possible interactions with milk bioactivity are endless and highly complex. Milk bioactive proteins affect health outcomes, and each health state influences how milk bioactive proteins work in the body.

Through the application of natural scientific methodologies, thousands of milk proteins with health effects beyond their nutritional value have been identified, in part by using proteomics technologies [104,105,106]. Some of these proteins (or protein groups) are shown in Figure 5 (left side). This list indicates the extreme diversity and complexity of milk proteins with presumed bioactive properties. The bioactivity of these proteins may be indicated by their physical or chemical structures, and, for some proteins, also by their documented health effects at cellular, tissue, or whole-body levels in (natural) scientific studies. However, for most milk proteins, their bioactive properties remain poorly understood at the physical level of health (Figure 5, right side). When possible health outcomes are expanded beyond physical health alone to cover social, mental, or spiritual health, it is clear that milk bioactivity is impossible to fully document at all health levels. The number of interactions between bioactive proteins and possible health outcomes is endless.

Strict confidence in reductionist, structure–function research when verifying milk bioactivity can be viewed as ‘nutritional scientism’ (Supplementary Figure S2, [57,108,109]). Milk bioactivity scientism shares similarities with faith, because bioactivity is impossible to test and verify at all levels of interacting milk bioactive proteins and health outcomes. Milk bioactivity and human health are study fields that span across many scientific domains, partly explaining why it is difficult to define milk protein bioactivity by science alone. The health effects of different proteins beyond natural science alone are unlikely to be additive, but may be antagonistic or synergistic, making such multidimensional health outcomes difficult to handle when using normal scientific methodologies. From the myriad of milk bioactive proteins and their two-way interactions in all four dimensions of human health, it is evident that natural science can provide ‘complete scientific evidence’ for only a few milk bioactive proteins. This conclusion contrasts with the science of pharmacology, often focusing on cell-specific, single molecules for disease treatments relating to specific body targets. Even if milk bioactive proteins are isolated and purified, their physiological actions on the human body may interact with many other milk components and body molecules, depending on the target cells, protein concentration, technical formulation, and specific health state of the host. Thus, the prediction that ‘science will answer all questions in the future’ is not realistic for milk bioactivity, especially not when using natural science alone.

In recent decades, omics technologies have been applied as ‘holistic’ add-on techniques to traditional (single-factor) milk bioactivity research in the natural sciences. Genomic analyses of milk and infant cells are used to identify the breast milk miRNA regulators that affect immune maturation [98], transcriptomics to assess the expressions of milk stem cells and host gene expressions [110], proteomics and peptidomics to characterize all milk proteins and host cell protein responses [111], epigenomics to explore the (longer-term) effects of milk on host gene expressions [112], metabolomics to assess interactions with milk or host metabolites [113], and microbiomics to assess the interactions between milk and host microbes [114]—just to mention a few examples. Such techniques, and the wealth of new data they contribute, provide a broader picture of milk components and their multiple effects on the human body. Yet, omics technologies remain poor tools when seeking clarity about specific mechanisms beyond valuable information about hitherto unknown components and their biological networks and connectivity. Omics analyses of biological samples reflect the status of the genes, epigenetic regulators, proteins, metabolites, or microbes at one site in the body, at one point in time. Therefore, omics technology applied to milk bioactivity science has markedly increased the total number of presumed milk bioactive proteins and their potential health outcomes. However, the application of omics technologies to milk bioactivity research has increased (not decreased) the number of unanswered questions and expanded the gap between the ‘known’ and ‘unknown’ in science.

Existential, spiritual, or religious forms of faith (Figure 1) cannot and should not fill the increasing knowledge gaps in science. Historically, faith-based convictions have been used to fill in knowledge gaps in science (‘God of the gaps’, [115]), yet faith-based convictions encompass more than filling knowledge gaps in science (see Section 3). Omics analyses show potential but also some limitations apparent in current milk bioactivity science and clinical therapy for infants [116]. Among these, proteomics fails to provide a more holistic and mechanistic picture of milk bioactivity, and proteomics data have highlighted how relatively little we still know and understand about human milk proteins [117]. Omics analyses are important in terms of finding new scientific questions and hypotheses, but seldom provide concrete answers to clinical questions: Which milk proteins do what, when, and how? How to feed infants? Access to tissues and cells in animal studies has enabled the study of tissue responses to milk interventions via multiple layers of transcriptomic, epigenomic, proteomic, and metabolomic information. Yet, the ability to translate this vast amount of information into clinical solutions for infants has proved difficult, especially when using highly controlled experimental conditions in animals [118,119,120,121,122,123,124].

## 7. Colostrum Bioactivity

Colostrum formation by the lactating mammary gland of mammals starts before birth and represents milk production during the first few days of life. Colostrum not only provides nutrients, but forms a critical ‘biological bridge’ to support the transition from the sterile life in utero to the microbe-rich environment of postnatal life [10]. In newborns, colostrum prepares the intestine to combat bacteria and tolerate the new environment while allowing for sufficient time to develop independent immunity. In many species, newborns are virtually 100% dependent on colostrum for protection and survival, while in other species, colostrum is important but not critical for health and survival (e.g., humans). Across species, colostrum contains higher amounts of milk bioactive factors than mature milk.

In some cultures, discarding colostrum has been practiced [125], but the biological and clinical importance of human colostrum has received much attention, especially for the most vulnerable, preterm infants. Recent meta-analyses and systemic reviews showed that even very small amounts of a mother’s colostrum reduce the incidences of colitis, sepsis, lung disease, feeding problems, and growth deficits [126,127]. These findings are supported by some reviews [128], but questioned in others [129], and the mechanisms underlying this are unknown. Consequently, we cannot say that we have obtained full scientific evidence for the health effects of colostrum. This would require that health mechanisms are documented in infant or animal intervention studies, connecting effects with specific milk ingredients and structural body responses. Until now, the clinical effects of colostrum have only been statistically documented in some but not all studies. The finding that even small amounts of colostrum are efficacious makes it likely that these effects are related to true ‘bioactivity’, beyond providing nutrients. Bioactivity may be derived from numerous interacting milk factors, but could be found among the hundreds of different proteins, stem cells, or small-molecule gene transcription regulators (e.g., miRNAs) [130]. Looking for a ‘needle(s) in a haystack’ is what best describes research focused on identifying the mechanisms involved in the health effects of colostrum. Until further evidence can be obtained, we have reasons to ‘believe’ that the statistical effects reported across studies are indeed true, despite the significant variations in results and lack of mechanistic evidence. The assumed value of colostrum for newborn mammals (especially those born preterm) is a rational understanding, belief, and common sense. Yet, this does not make the health effects of colostrum a fact proven by science. Enriched amounts of immunoglobulins in the colostrum are likely to be only a small part of the scientific explanation for its effects.

Human colostrum is often not available from the mother after the delivery of a preterm infant, making it necessary to look for supportive therapies derived from animal colostrum. Across mammals, colostrum shows remarkable compositional differences, but it always contains high amounts of milk bioactive proteins. Numerous review papers on the clinical use of bovine colostrum have been published in recent years [10,25,131,132,133,134], yet the number of remaining questions is steadily increasing as we apply an increasing range of scientific methodologies to study colostrum’s bioactivity, especially at the level of natural science. Bovine colostrum continues to be widely used as a health food supplement for immune support in healthy adults, athletes, and patients with gut and systemic infections, skin diseases, and some cancers [135]. We recently reviewed the use of bovine colostrum in pediatrics [10], and across clinical studies, the suggested beneficial immune effects of bovine colostrum were difficult to demonstrate when looking at evidence from the clinical studies. Thus, efficacy in terms of scientific evidence alone does not seem to determine whether colostrum or fractions thereof are being used at the individual or societal levels. There are great variabilities among the available bovine colostrum products [136], partly due to variable industry treatments and fractionations (destroying parts of its bioactivity). In the public, knowledge from science appears to be combined with faith convictions to determine understanding and common beliefs regarding the health effects of colostrum.

Considering the myriad of (interacting) milk protein bioactives in colostrum, it is not surprising that complete structure–function mechanistic evidence for health effects is difficult to obtain, even when using a combination of chemical, cell, and animal studies. However, it is surprising that human trials, after much support from cell and animal research, often fail to demonstrate clear clinical benefits. Our own attempts to document that bovine colostrum benefits preterm infants lacking human milk illustrate this controversy. Chemical and cell studies have proven that intact, unfractionated bovine colostrum contained numerous protein bioactives, even after spray-drying and heat pasteurization, which inhibited bacterial growth and exerted immune modulation [22,31,137]. Numerous studies in preterm pigs, used as models for infants in different clinical conditions and product combinations, demonstrated clear benefits for the gut, immunity, brain, and metabolic outcomes relative to infant formula [10]. Following our own infant pilot trials [15,17,18] and others in India [138] and Egypt [139], the results of much larger trials on preterm infants (total *n* = 700) at hospitals in Denmark and China were recently reported [5,13,29]. The results showed no effects or inconsistent effects of colostrum supplementation. We reached similar conclusions in trials on supplementing bovine colostrum for infants with short-bowel syndrome [15] or chemotherapy-induced mucositis [25]. Do such results mean that the safety, efficacy, or harm of bovine colostrum for infants have been proven or disproven? Neither. Further properly designed clinical trials are required to account for all the new questions arising from previous trials. Until then, we have to rely on what we ‘understand’ and ‘believe’ with regard to the clinical use of bovine colostrum for infants. Contributing to such combined beliefs are the results of social and human science studies evaluating health economics, cultural/religious acceptance, and the ethics of using bovine colostrum for preterm infants. The final decision of hospital personnel and parents to use bovine colostrum for individual preterm infants (when human colostrum is absent) may be influenced by personal faith-based convictions and philosophical/religious/cultural concerns about milk kinship. The clinical practice of colostrum feeding for newborn infants is, thus, based on scientific evidence from product, cell, animal, human, and societal studies in combination with communal or personal faiths, together forming agreed-upon beliefs and guidelines (Figure 1).

## 8. Infant Formula Bioactivity

Infant formula is required when maternal milk and breastfeeding is not available or possible. Formula feeding is a highly sensitive topic that may involve science, belief, and faith perspectives. This extreme sensitivity can be observed in current public debates on breastfeeding policies and attitudes relating to infant formula production. The formula industry still suffers from a bad reputation due to allegations and concerns related to its marketing practices in lower- and middle-income countries about 50 years ago, particularly in Africa. The controversy focused on accusations that the aggressive marketing of infant formula was undermining breastfeeding practices and contributed to infant malnutrition and even mortality [140]. The melamine milk scandal in China is another example of the far-reaching public consequences of the adverse effects of manipulating formula for infants [141]. Milk and infants are sensitive topics, affected by scientific knowledge but also by beliefs and faiths (fear).

Research projects on infant formula often involve industrial co-sponsorship. The majority of formula products are based on cow’s milk proteins (variable mixtures of casein and whey), plus vegetable oils to replace dairy fat for both nutritional reasons and product stability. The recent resistance to accepting the use of cow’s milk products for infants, especially for the most vulnerable preterm infants, is based on firm scientific evidence relating to the adverse clinical effects of formula [99,142]. The exact biological mechanisms behind such adverse effects remain unclear, but the lack of milk bioactives that are normally present in human breast milk is thought to be a determining factor [143]. In addition, it is possible that formula products do not contain the appropriate amounts or right balance of the multitude of different nutrients present in fresh breast milk. However, there is no firm evidence to support that formula based on cow’s milk is better or worse in terms of allergies in infants than milk products from other mammals (e.g., goats, sheep, buffalos, camels, horses, and donkeys), despite the fact that compositional differences affect allergy sensitivity [144]. Specifically, for preterm infants, donkey and goat milk supplements have been tested to investigate if the milk nutrients and bioactives from these species protect against allergies, maldigestion, and dysmetabolism when compared with cow-based supplements [145]. Evolutionary theory would suggest that both nutrients and bioactives are closely adapted to the growth and development of each species. On the other hand, health-promoting bioactive components may act both within and across species.

Many heat-labile milk protein bioactives are denatured in infant formula production due to the need for fractionation and heat treatment procedures to adjust its composition and ensure its microbiological safety. The carbohydrate and lipid constituents in milk are less sensitive than many intact proteins. Much research has been undertaken to adjust these carbohydrate contents via the addition of specific oligosaccharides that may mimic the assumed beneficial effects of vast amounts of oligosaccharides in human milk (more than 100 different components). Isolated bioactive milk proteins (see Figure 2 and Figure 5) have not been tested as supplements for formula to the same extent, partly due to their high sensitivity to degradation, challenges related to their isolation and stability, and possible risks for side effects. Instead, much research focuses on reducing the possible adverse effects of milk processing, as protein damage related to the many processing steps may affect biological responses, especially for preterm infants. Processing and storage effects, even from single-step heat pasteurization, are believed to be a key reason why human donor milk remains inferior to a mother’s own milk for preterm infants [33]. When using preterm pigs as a model for infants, the gut-protective effects of whey protein in formula appeared to decrease as the intensity of heat treatment steps increased [6,32,33,36], especially when using ultra-high-temperature (UHT) treatments and long storage times to produce ready-to-feed (RTF) liquid formulas [30,146]. While heat damage to the protein fraction is well known, the mechanisms whereby this affects the immature gut are unknown.

Much formula research has focused on ‘humanizing’ cow-milk-based infant formula, e.g., making its composition more like the known constituents present in human milk [143,147]. This is based on the belief (or common sense) that human milk composition, although highly variable, is well adapted to suit the needs of infants. Mammalian evolutionary theory may even support that mammalian milk is closely adapted in its composition to the needs of individual infants, supporting individual mother–infant dyads. While this is a reasonable belief, this cannot currently be known or tested with any certainty. Especially for infants with special needs (e.g., preterm infants), it is widely accepted that a mother’s own milk is not sufficient as the sole source of nutrients and protective factors. Second, the great variability in milk nutrients and bioactive factors among mothers and lactation stages suggests that a very close ‘match’ between infants and their own mother’s milk is unlikely. Third, it is simply impossible to make all the components in cow’s milk formula similar to those in human milk. Specifically for protein components, many of these would be highly species-specific. Therefore, a fully ‘humanized infant formula’ not only needs to have its concentrations adjusted for multiple components, but would also need to modify the structures of many milk proteins. This is obviously not possible, making the idea of producing humanized formula based on natural scientific knowledge more a matter of faith than a matter of science. Faith convictions related to humanized formula relate mainly to the production of premium, high-price formula products, yet these ideas of producing near-human milk formulas also influence the public understanding of and attitudes towards mainstream infant formulas. The marketing, economic, and commercial aspects of humanized infant formulas are studied in the social sciences. The assumed superior ‘naturalness’ of some formula products versus others, and the ethical dimensions of this, are study topics in human sciences. In addition to knowledge from these three science domains, this paper argues that faith convictions, which go beyond scientific knowledge, also play a role in the public understanding of the positive and negative health effects of formula feeding for infants.

## 9. Bioactivity of Isolated Milk Fractions or Proteins

In the sections above, it is highlighted that the complex composition (proteins and many other components) of intact milk and colostrum, or fractions thereof, makes it difficult to verify the mechanistic relationships involved in milk bioactivity, not only when studied in humans (only allowing for clinical effect analyses), but also when using animal model studies (allowing for detailed organ insights, plus multi-omic analytical depth). The bioactivity of isolated milk fractions can most easily be documented using cell studies, but the translational value in the human clinical situation is difficult. Across all models, the fact remains that the number of possible interacting protein components in milk fractions is too many to allow for detailed cause–effect analyses. An additional complication in detecting the health effects of milk bioactives is their variable oral intake, together with milk diets or other diets beyond infancy. This complicates the interpretation of the bioactivity of specific proteins in food matrices, with variable effects of digestion and microbial exposure in the gut and minimal absorption of intact milk proteins into the bloodstream. Together, these limitations indicate that most bioactivities of milk proteins are caused by local effects in the gut [148]. Gastrointestinal survival is required for milk bioactive proteins to exert such physiological effects in the gut, although some may also act via their peptide degradation products [49]. The effects of milk proteins beyond the gut would be indirect, with or without direct effects on distant organs, such as the brain and internal organs. Provided that such proteins can be isolated from bovine milk and are proven safe in humans, they can potentially be used to enrich infant formula with bioactivity. From these perspectives, the effects of a few well-known milk bioactive proteins are described below.

The current understanding of infant formula supplementation is based mainly on knowledge from natural science (e.g., regarding composition, safety, and efficacy at the product and body levels). However, social science (e.g., societal, legal, and economic constraints) and human science (e.g., history, ethics, and value of supplementation) also contribute to form the current beliefs regarding specific formula supplementations. In some cases, scientific evidence from all three domains of science is supplemented with faith-based convictions to reach beliefs that lead to safety guidelines and recommendations for the supplementation of formula or human donor milk. However, contributions from faith convictions to understand the effects of isolated milk proteins are likely fewer than those for milk fractions, intact milk, or colostrum products due to the complex composition of the latter products, making scientific interpretations difficult. Conversely, controlled research on isolated, well-defined proteins from milk is a relatively exact natural science, with similarity to pharmacology research using cells, animals, or humans to investigate the detailed biological health effects of highly specific, well-defined single molecules.

Milk Fat Globule Membrane proteins (MFGM, <5% of total milk protein) have received considerable interest as proteins derived from milk, and their bioactive properties have been demonstrated across cell and animal studies. The MFGM protein fraction is a highly diverse group of proteins, not a single protein, covering >1000 different proteins, as shown via proteomic analyses [105]. Different MFGM proteomic studies have shown widely different numbers and amounts of proteins due to differences in the isolation techniques used, purity, and natural variability among milk samples. The enrichment of infant formula with bovine MFGM showed a marginal improvement in immunity and cognitive parameters, but the affected parameters were only few among many endpoints tested [149]. Combined with results from other studies [150,151,152], the evidence in favor of bovine milk MFGM supplementation remains weak. Yet, bovine MFGM is now believed to be safe to add to formulas [153,154]. Safety is critical, but the role of future science will be to prove clinical efficacy, as well as mechanisms for different groups (e.g., preterm/term, age, gender, and ethnicity). Realistically, it is not possible to perform all of the required scientific studies to obtain full evidence for both safety and efficacy.

Immunoglobulins (Igs) are most abundant in colostrum, but are also found in small amounts in mature milk. They exert local microbial protection in the gut (not only IgA, but also IgG), and these bacteriostatic and immunomodulatory effects may work across species [10,134,135,155]. The local gut-protective effects of bovine IgG have motivated studies on bovine colostrum as a supplement for preterm infants [5,13,29,156]. Isolated bovine IgG has been tested in a series of human studies, with promising effects being noted in relation to microbial colonization and gut infections [157]. However, when pure human Igs were added to the diets of breastfed preterm infants, the effects on feeding intolerance and necrotizing enterocolitis (NEC) were marginal or absent [158]. Possibly, the effect of the addition of purified Igs is limited when supplementing breast milk that already contains high amounts of IgA and other gut-protective proteins. Until now, the dietary intake of purified fractions of human or bovine IgG has not been used in clinical practice, despite natural scientific research indicating their safety and efficacy.

The whey protein α-lactalbumin is already extensively used in formula production for infants, partly for nutrition and partly for bioactivity reasons. Relative to the most abundant protein in bovine milk, β-lactoglobulin, α-lactalbumin has an amino acid composition more similar to that seen in human milk (essential amino acids and branched-chain amino acids—BCAAs) and is believed to be the optimal protein source for infants and children. Hence, the supplementation of α-lactalbumin in infant formula is widely used to ‘humanize’ infant formula [159]. Whether α-lactalbumin has specific health bioactivity beyond nutrition (e.g., gut, immunity, or brain) is unclear, and neither animal nor infant studies can confirm this [7,159]. Confirmed cow’s milk allergies are just as often induced by immunological hypersensitivity to α-lactalbumin as by more dominating bovine milk proteins like caseins and β-lactoglobulin [160]. Hence, the benefit of α-lactalbumin-enriched formula relies on the sensible but controversial idea that an excessive supply of amino acids is detrimental and predisposes to obesity, despite the fact that many other factors also play roles [161]. Natural scientific evidence, direct and indirect, in combination with sensible beliefs, determines the guidelines and current clinical practice for the enrichment of infant formulas with α-lactalbumin.

Similar to the case of α-lactalbumin, the relatively low concentrations of the multifunctional peptide osteopontin in bovine milk have stimulated speculations on supplementing infant formula with this peptide based on osteopontin isolated from bovine milk [162]. The bioactivity of osteopontin of both human and bovine origin can be clearly demonstrated in gut cell studies in vitro [163], while more moderate and variable effects are found along mucosal surfaces in animals [12,22,164]. For this peptide, like for many other regulatory peptides, the fact that osteopontin is produced endogenously by many cells in the body (not only mammary gland cells) complicates its interpretation. Somewhat surprisingly, dietary osteopontin increases the expression of brain osteopontin in young mice [165]. The fact remains that ‘humanizing’ infant formula with osteopontin relies on the belief that a certain osteopontin level in milk is beneficial for infant health, if not across the body, then at least locally in the gut. While this is a reasonable belief from an evolutionary perspective (‘evolutionary common sense’), it is difficult to prove or falsify using natural, social, and human sciences due to the numerous interacting variables between milk proteins and human health outcomes (Figure 5).

Lactoferrin is an important iron-carrying and antimicrobial protein in milk, but is also found in many other body fluids. The relatively low lactoferrin concentration in bovine milk, especially after processing, has led to attempts to humanize infant formula by adding lactoferrin, especially for infection- and gut-sensitive preterm infants. Numerous studies on bovine lactoferrin have been carried out in cells, animals, and humans, mostly with beneficial effects. Yet, the addition of bovine lactoferrin to infant formula is not widely accepted in clinical practice. Increasing the price of formula is a natural limitation, but the main problem relates to the inconsistent effects across studies or even the risk of over-supplementation [34,37]. In the largest infant study to date (*n* = 2200), bovine lactoferrin did not improve resistance to gut disorders or infections in preterm infants [166]. This result was disappointing, considering the vast efforts invested in such ‘definitive high-power trials’. Such outcomes raise concern about the value of clinical trials and question the scientific methods currently used and their ability to prove the effects of single dietary bioactive peptides in clinical settings with multiple interacting variables.

Insulin-like growth factor 1 (IGF-1) has received a lot of attention as a possible supplement in infant formula. Other milk proteins in the same category are transforming growth factor β (TGF-β) and epidermal growth factor (EGF) peptides. There is little doubt that any effect of dietary supplementation has only local gut effects, as the peptides cannot be absorbed, or can only be absorbed in small amounts, relative to the release of the corresponding endogenous peptides produced by many cell types. Specifically, if dietary growth factor peptides can escape digestion, which is likely in the low-proteolytic environment of newborn infants, then there is theoretical potential to improve the functionality of digestive, endocrine, immunological, and neurological cells in the gut. Nevertheless, the oral administration of these peptides has not reached clinical use, not even for the most vulnerable (preterm) infants without access to their mother’s milk. Again, an important reason may be related to the inconsistent effects reported in human infant trials [167,168] relative to the numerous supportive cell and animal reports that have demonstrated positive effects of a systemic or enteral supply of IGF-1 [169,170], TGF-β [171,172], or EGFs [41,173] in formula. Enriching a diet with bioactive components that are already widely produced endogenously in the body may explain the disappointing effects of IGF-1 studies, together with its poor absorption into the bloodstream. Another reason may be limitations due to a too reductionist understanding of how a single growth factor may act in the body, not fully acknowledging the immense complexity of varied cell and tissue responses, interactions with other regulators, and with other dietary components given together with IGF-1.

## 10. Science, Faith, and Breastfeeding

**Figure 6:**
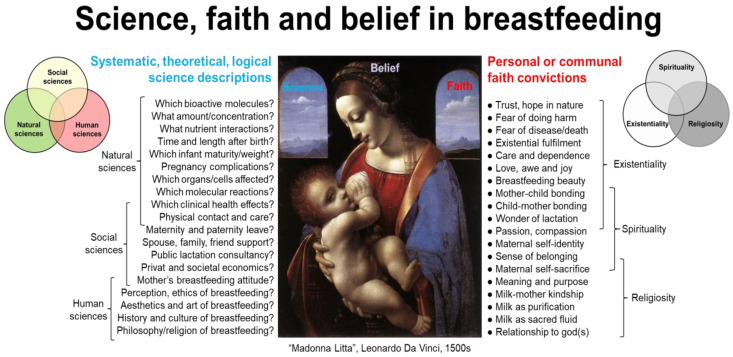
Scientific research into the bioactivity of breastfeeding and human milk for infants requires natural, social, and human sciences to answer all relevant scientific questions. The (left side) of the figure provides examples of such questions and the corresponding science field(s). Scientific evidence is incomplete, in part due to the multitudes of interacting variables and difficulties in performing randomized, controlled studies on mothers and infants. Physical, social, and personal benefits of breastfeeding for mothers and infants are well documented by clinical science, but cause–effect relationships and molecular mechanisms (natural science) are unclear. Faith elements, including existential, spiritual, and/or religious attitudes to breastfeeding and human milk may antagonize, complement, or synergize with knowledge from science to form the beliefs that are the basis for public understanding, guidelines, and practice. Examples of faith convictions indicating existentiality, spirituality, or religion, or a combination, are shown in the figure (right side). Looking into breastfeeding and human milk from both science and faith ‘windows’ facilitates a broader and more nuanced picture of breastfeeding than from the science perspective alone. Image from WikiMedia: ‘Madonna Litta’, attributed to Leonardo da Vinci (1452–1519), Italian scientist, naturalist, and artist.

Breastfeeding offspring with maternal milk is a core characteristic of mammals. Breastfeeding connects the newborn infant intimately to its mother, physically and socially, allowing for the direct passage of milk as the exclusive diet in the first months of life, with the need for close physical and mental cooperation between the mother and newborn infant. From an evolutionary perspective, it is reasonable to claim that breastfeeding with human milk and its many bioactive constituents must be critical for infant health, even without full evidence from science. However, ‘common sense’ does not suffice as scientific proof for breastfeeding. Breastfeeding is often termed ‘the gold standard’ of infant feeding [142], and this belief is based partly on scientific knowledge and partly on faith convictions. ‘Faith in breastfeeding’ covers strong personal and emotional opinions about the value and practice of breastfeeding, leading some to denote mother’s milk as ‘magical’ [174]. Uncertain or incomplete scientific evidence is coupled with aspects of meaning, hope, joy, and fear related to breastfeeding, together forming the accepted belief that ‘breast is best’ for infants [175]. Using a classical piece of art, Figure 6 illustrates how beliefs in breastfeeding for infants are formed not entirely by knowledge from science, but also by such faith-based convictions. Science asks open questions to provide new knowledge based on data collection and systematic, logical reflections (left side, Figure 6). Faith entails personal or communal convictions that go beyond logical reflections and truths revealed by science (right side). Some elements of each are described below as the last example of how science interacts with faith to determine our beliefs and understanding of milk bioactivity.

The clinical benefits of breastfeeding are well documented in the scientific literature, albeit not in terms of mechanistic, physical, and natural science, but mostly by human observational studies that can be considered as a social science (or a mixture), according to the methodological criteria laid out for each science (Supplementary Figures S1 and S2). Strict natural scientific evidence of breastfeeding requires that health mechanisms are documented in milk intervention studies and that their effects are connected with specific milk ingredients and structural body responses (e.g., tissues, genes, proteins, metabolites, and hormones—Figure 6). Unsurprisingly, complete answers to such detailed questions are impossible to find, considering the vast amount of milk bioactives, multiple short- and long-term health responses, and their many interactions (Figure 5). Intact human milk or specific milk bioactive proteins affect developing cells in the gut, cardiovascular system, liver, immune system, and brain in vitro [2,48]. While such studies support the beliefs in breastfeeding, they do not prove that specific milk bioactives are responsible for the protective effects of breastfeeding. Further, most clinical studies on infants report only associations, not cause–effect relationships between breastfeeding and health outcomes. Further, the number of milk bioactives and possible health outcomes are too many to study (Figure 5) across the many cell and tissue types in infants or animal models. Finally, there are numerous conflicting results in the field, limiting natural science from being the only source of valid information to form beliefs that lead to public policy and understanding. It is, thus, impossible to maintain a position of strict ‘nutritional scientism’ [57,108,176] in relation to the health effects of breastfeeding, based on existing scientific knowledge.

Social and public health sciences have documented the clinical effects of and answered questions about how breastfeeding relates to motherhood, social relationships, society, economics, and politics (Figure 6, left). In the early 20th century, there was a shift away from breastfeeding in some high-income countries due to the promotion of formula feeding and women entering the workforce. However, as scientific research progressed and more evidence emerged concerning the clinical benefits of breastfeeding, policies changed. At the population level, there is no longer any doubt that breastfeeding is clinically superior to formula feeding. The benefits are greatest in the first year or two of life, especially for infants with special needs (e.g., preterm infants [177]). The documented benefits for normal infants are less, but are highly statistically significant across large population studies, as recently shown in South Korea [178,179,180,181]. Based on such studies, the WHO recommends exclusive breastfeeding for the first six months of life and has published clear policies on this in many settings [182]. These recommendations are based mainly on the anticipated physical health effects of mother’s milk (bioactivity), not on providing particular nutrients from milk (which could, in principle, be derived from other food sources). Yet, such authorities acknowledge that the overall scientific evidence for breastfeeding from natural science and clinical trials is moderate or even weak [182].

Public policies on breastfeeding are co-determined by answers to questions from social and human sciences, going beyond physical health and nutrition (Figure 6, left side). While natural science has contributed knowledge about the chemical contents of human milk and physiological responses in infants (or animal models), social sciences have contributed knowledge about clinical effects together with social, legal, economic, cultural, and political factors that are important for breastfeeding. Finally, human science produces systematic knowledge about many philosophical, aesthetic, and ethical aspects of breastfeeding [183,184]. Human sciences conduct descriptive, theoretical studies on motherhood and breastfeeding, including ethics [185] and its perceived ‘naturalness’ [186], but the descriptions do not by themselves provide meaning, value, and hope to individuals, groups, or society. Social sciences combine collected data and human reflections on breastfeeding to study topics such as gender issues, the labor market, and health economy in relation to breastfeeding [187,188,189]. This tension between the theory of breastfeeding from all the sciences and the practice of breastfeeding (partly based on faith convictions) together form the beliefs and understanding that lead to guidelines.

Figure 6 (right side) illustrates that practical breastfeeding can be associated with faith-based existential meaning for mothers, infants, families, and society. Such faith convictions may relate aspects of love, duty, and communal suffering with the newborn child, reflecting some core human values and search for meaning across many life contexts [65]. General faith in nature and the intricate and beautiful evolutionary adaptations in the natural world (mother, birth, infant, and milk) are other elements within secular ‘existentiality’ related to breastfeeding. Love, passion, and compassion for mothers, infants, and their intimate nutrition–health connections are components that are difficult or impossible to characterize or validate by science. Nonetheless, these elements are co-drivers for beliefs and understandings related to breastfeeding. On the negative side, fear of infant disease and mortality may plays a strong role in faith convictions and maternal psychological distress [190], regardless of scientific knowledge. Strong faith convictions in the naturalness and moral necessity of breastfeeding, with or without spiritual or religious perspectives, pose a risk for superstition, unfounded beliefs, and misguided practice. Thus, unfounded beliefs in certain breastfeeding practices, requiring strict maternal behaviors and diets, have the potential to negatively impact mothers, infants, and public policy.

There are overlaps between strictly secular faith-based positions concerning human meaning, existence, and value and those that, to variable degrees, involve attention to transcendent powers or God(s), religious institutions, and dogma. There are many examples of ‘faith in breastfeeding and mother’s own milk for human infants’ receiving inspiration from spirituality or religion (see Section 5, Milk Bioactivity in Religions). In both high-income and low- and middle-income countries, spiritual and religious practices influence breastfeeding choices or choices concerning the use of donor human milk or milk from other species [191,192,193]. These faith-based influences may antagonize, complement, or synergize with evidence produced from science. Sciences should avoid pseudoscience and scientism by attention to the uncertainties and inevitable unknowns in science [57,58,108,176,194,195,196]. Faith perspectives should avoid superstition by attention to correction by scientific knowledge ([58,197], Supplementary Figure S2). This perspective paper contributes to solve these difficult tasks.

An example of conflicting evidence related to breastfeeding is its effects on infant infections and allergies [198,199]. As an evolutionary benefit, breastfeeding may protect against infections, while effects against allergic diseases are more questionable, or at least depend on many factors, including hygiene standards. Scientific controversies in the field may have scientific reasons (e.g., inadequate control of interactions with genetics, microbe exposure, diet, and diagnostic variability), but could also relate to factors beyond scientific investigation, including what mothers believe they should do [200]. While this conclusion does not challenge current convictions about the benefits of breastfeeding, it calls attention to the need to avoid both academic scientism and superstitious faith in milk bioactivities (Figure 1, Supplementary Figure S2). Both scientism and superstition related to milk bioactivity carry the risk of supporting breastfeeding policies with false arguments. Faith in the health benefits of breastfeeding is reasonable from the perspective of mammalian evolution, known biological mechanisms, social functions, and humanistic understanding of humans, nature, and mammals. Thus, science and faith elements can synergize to form the beliefs that inform the personal and public understanding of breastfeeding.

## 11. Conflict or Synergy between Scientific Knowledge and Faith Convictions

Science and faith perspectives of milk bioactivity can be in conflict with each other. In relation to milk bioactivity, it is critically important that strong faith-based convictions (secular or spiritual/religious) about breastfeeding, infant formula, or milk bioactive proteins do not overrule empirical data, logical reflections, and scientific knowledge. A historical example of science–faith conflicts from biology was when claims of René Descartes (1596–1650) about the human soul being placed in the pineal gland of the brain were rejected based on empirical, anatomical observations by Niels Steensen (1638–1686) [201]. Steensen also showed that the human heart was ‘only’ a muscle and not the seat of human passion and warmth (faith), as suggested by Descartes [202]. While milk is ‘only’ a body fluid with special constituents and special properties, this paper suggests that additional perspectives are important. Science and faith represent complementary and, to some extent, synergistic ways to understand milk bioactivity. Science and faith concepts can cross-fertilize to reach more complete views of milk than those possible by science alone. This perspective makes it relevant to understand milk bioactivity not only in terms of what can be explained by available scientific knowledge, but also by faith-based elements covering existential meaning, experience, spirituality, or religiosity (Figure 1).

Both belief and faith support the science of milk bioactivity by retaining an epistemological condition of doubt and uncertainty in the observable [47]. What is seen (and measured by numbers, detected by structures, and defined by logical arguments) does not reveal all there is. Rather than inhibiting science, faith may stimulate scientific curiosity and fuel further investigation, like for many of the founders of natural science and biology, from Newton, Bacon, and Descartes to Steensen [203,204,205,206]. The observed bioactivities exerted by milk proteins open up an ever-increasing sea of knowledge, where mechanistic structure–function relationships, together with social and human science descriptions, explain part of the whole. Faith-based convictions add existential, spiritual, or religious perspectives expressing meaning, hope, love, or fear in relation to milk and infants (Figure 6). Faith helps to acknowledge and appreciate the strong symbolic value of milk in many cultures and some religions as a matter of spirituality that is, at least partly, beyond detailed scientific inquiry. Faith-based understanding may facilitate the use of milk or milk products for infant health. Faith-based perspectives help to avoid undue milk bioactivity scientism and faith does more than fill in the gaps of science. Together with science, faith convictions form what we believe and understand about milk bioactivity, providing a foundation for how to use milk bioactivity to maximize infant health and well-being.

Science supports faith with data, systematic knowledge, and logical arguments to avoid misconceptions and superstition about milk bioactivity, as well as fear of milk from other species and infant formula. Scientific discovery stimulates awe, wonder, and respect for nature’s intricate structures and meaningful relationships. If such faith stimulated by scientific discoveries involves spiritual or religious elements, then further scientific discoveries have the capacity to correct or complement previous faith convictions. Science helps to set boundaries for faith-based approaches to milk bioactivity (e.g., milk rituals and healing, species-specificity of milk, mother–infant dyads, cross-fostering, milk kinship, formula feeding, and dairy product skepticism).

Holistic, science-, and faith-based approaches to milk bioactivity may increase the focus on milk as a complete product rather than excessive attention to separate milk proteins, supporting that the ‘whole is more than its parts’ in biology and medicine [207]. Faith perspectives help in accepting the fact that the clinical (safety) effects of milk bioactivity can be proven statistically, but that it is difficult or often impossible to disentangle the mechanisms of how they work in different human conditions. The multitudes of bioactive milk proteins (interacting with myriads of carbohydrates, lipids, minerals, and vitamins) likely work together, not independently. When such complex interactions among milk constituents are coupled with even more complex health responses (physically, mentally, socially, and spiritually, Section 4 and Section 6), this indicates that full understanding can never be reached. It also reduces enthusiasm for advocating for one more large human intervention trial that will provide firm and lasting answers to each specific question concerning the bioactivity of milk (Section 9). Thereby, faith-based perspectives to milk bioactivity lend more support to large-scale observational studies (indirect detection of causation despite difficult control of numerous confounding variables) than to smaller, setting-specific intervention trials on humans.

Epidemiological studies assessing the post hoc effects of new health interventions reflect the scientific philosophy behind ancient health systems in China and India (Traditional Chinese Medicine; Arjuveda; Siddha Medicine [208,209,210]). Systematized knowledge from history, practice, and experience (a form of ‘statistical’ knowledge) is compiled into specific treatment remedies and forms the agreed ‘belief’ regarding safety and efficacy—until further evidence adjusts such beliefs. Based on our previous definitions of belief versus faith, these systems rely on a combination of both and add elements of modern medical sciences for diagnostic and preventive purposes, rather than therapy [211]. The ontological categories in these systems to understand nature, food, and human health (e.g., hot/cold foods, five-element theory, and energy medians) are used to form medical strategies based on ‘rational beliefs’, until new observational or scientific evidence points in another direction. Elements of faith (meaning, hope, trust, passion, and love) do play a role here in the overall view towards nature’s complexity and the need for the holistic preservation of biological integrity at both the food and body levels.

A respectful, humble attitude to the immense complexity of milk molecular structures, and their interactions with human health outcomes stimulate (rather than inhibit) empirical research and logical scientific reflection. ‘Reproducibility crises’ across many biological sciences suggest a need for more humility in science in terms of methods, data, and bias [44,45]. It is common to claim that science and faith should be kept separate. Yet, numerous modern scientists appear to embrace both contested scientific knowledge and faith convictions (existential, spiritual, or religious) when researching natural phenomena [212,213,214]. ‘*Beauty is that which we see, more beautiful is that which we know, but the most beautiful is that of which we are yet ignorant’* (Niels Steensen, 1638–1686, theologian and a founding scientist in anatomy, geology, and paleontology [201,205,215]). This humble statement is relevant for both scientists and the public when they, together, reach joint beliefs to try and understand and implement milk bioactivity for infant health.

## 12. Conclusions and Perspectives

Both science and faith can contribute to understanding milk bioactivity, albeit from very different perspectives and approaches. Science, as a systematic and evidence-based inquiry, plays a crucial role in unraveling the complexities of milk bioactivity at the levels of biological, social, and human science. Natural science employs rigorous methodologies to study the chemical composition, molecular structures, and biological effects of milk components, including their many different proteins. Social sciences show how milk bioactivity can be used at the social and societal levels, including dimensions of economics, law, anthropology, and politics. Human sciences may describe milk bioactivity from historical, philosophical, aesthetic, and ethical perspectives. Together, the latter sciences may also describe and discuss personal and communal beliefs related to milk bioactivity, including the glorification of human milk and breastfeeding, fear of formula feeding, gender norms, and motherhood. Through empirical observations, experiments, data analyses, and logical arguments, science uncovers not only biological mechanisms, but the wider role of milk bioactivity in human health. Together, natural, social, and human sciences elucidate the pathways through which bioactive molecules exert their context-specific effects and assess the safety, efficacy, and value of milk. Science provides a foundation of knowledge and theories that are constantly expanding and evolving through ongoing systematic research, experimentation, and logical arguments.

Faith convictions contribute to our understanding of milk bioactivity at a more practical level by adding broad perspectives of personal or communal experience, existentiality, spirituality, or religiosity. Approaches to milk bioactivity from a faith-based standpoint may attribute the bioactive properties of milk to divine providence, order, and interconnectedness in nature. Faith can influence personal perspectives on health and well-being and guide individuals to view milk bioactivity as part of a larger natural and social holistic framework. It can inspire beliefs in the healing power of nature or the spiritual significance of foods, including milk. For some, faith may provide a sense of meaning, purpose, and connection to the natural world. While science relies on empirical evidence and the systematic collection of data with logical arguments, faith involves convictions that transcend empirical and logical verification. Science explains the “what” and “how” of milk bioactivity, and faith elements provide insights into the “why” of milk bioactivity from a greater existential perspective. Via different paths, science and faith synergize to reach public beliefs. Science and faith are not ‘non-overlapping magesteria’ [216] to understand milk bioactivity. Rather, they hold the potential for mutual inspiration, honesty, humility, and synergy in finding practical solutions for milk bioactivity in infant health.

## Supplementary Materials

The following supporting information can be downloaded at: https://www.mdpi.com/article/10.3390/nu16111676/s1, Figure S1. Characteristics of scientific domains, as divided into natural, social and human (humanistic, humanities) science. Figure S2. Schematic presentation of overlapping insights into milk bioactivity from science, belief, and faith perspectives.
