# Supplementary material for: Science and Faith to Understand Milk Bioactivity for Infants
Source: Nutrients. 2024 May 29;16(11):1676. doi: 10.3390/nu16111676 (PMC11174769; doi:10.3390/nu16111676)
Supplement: Supplementary file 1 [file nutrients-16-01676-s001.zip › nutrients-2982690-supplementary.pdf]

nutrients MDPI

## Supplementary Figures

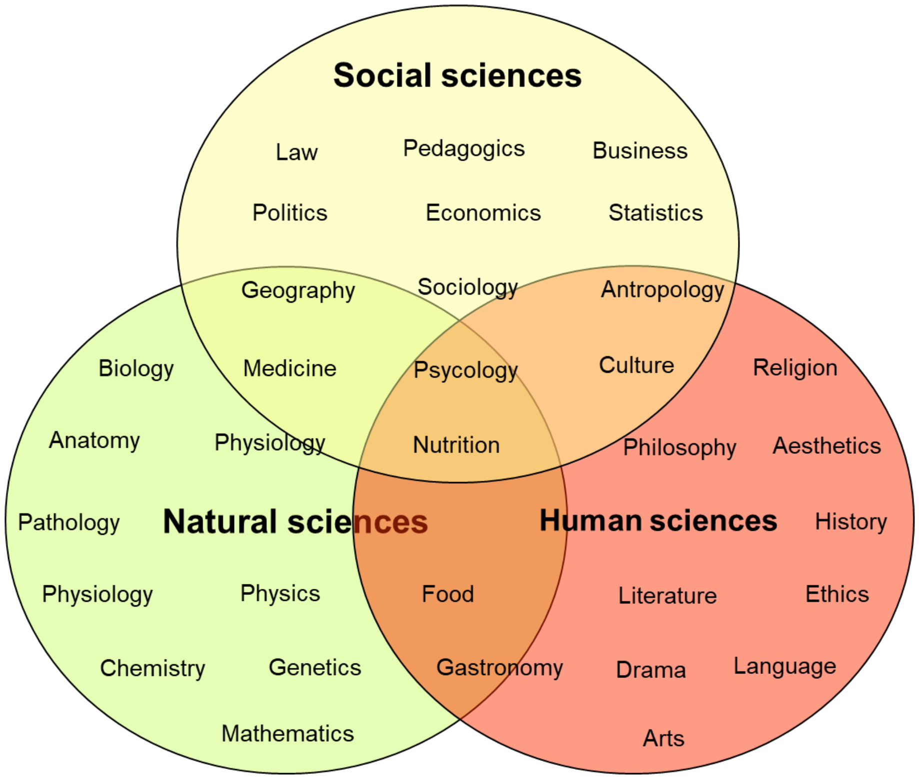


# Natural, social and human science characteristics

| | Scientific target | Study field examples | Time focus | Scientific methodology |
|---|---|---|---|---|
| **Natural science** | Description and control of the structure or function of material and physical objects in nature | Physics, chemistry, green environment, globe, plants, animals, humans, motion, molecules, mathematics | Past | Experimental, quantitative falsification/verification, using hypotheses, material mechanisms, reductions |
| **Social science** | Description of societal or human organisation, behavior and products. Material or immaterial. | Social structure, material production or distribution, politics, law, sociology, economics, anthropology | Past, future | Descriptive observations, statistical analyses, context and socially-dependent. Quantitative or qualitative |
| **Human science** | Description or evaluation of human acts, thoughts, feelings, culture, values | History, culture, ethics, literature, art, aesthetics, psychology, philosophy, , religion, language | Past, present, future | Qualitative descriptive, analytical interpretation, using logics, evaluation, theories and philosophies |

**Supplementary Figure 1.** The term "science" is in this perspective paper referring to all the academic activities at contemporary universities (e.g., the continental European definition of science), and relates to how topics and methodologies are divided into faculties. Further insights into the characteristics, similarities, and differences can be found elsewhere. Importantly, these three classical scientific domains show overlapping characteristics. Many study topics do not clearly belong to one of the three classical domains, but use research methodologies that relate to two, or even all three domains (e.g., topics like health, psychology, and ecology). The figure and table are constructed by the author based on general web sources and personal experience.

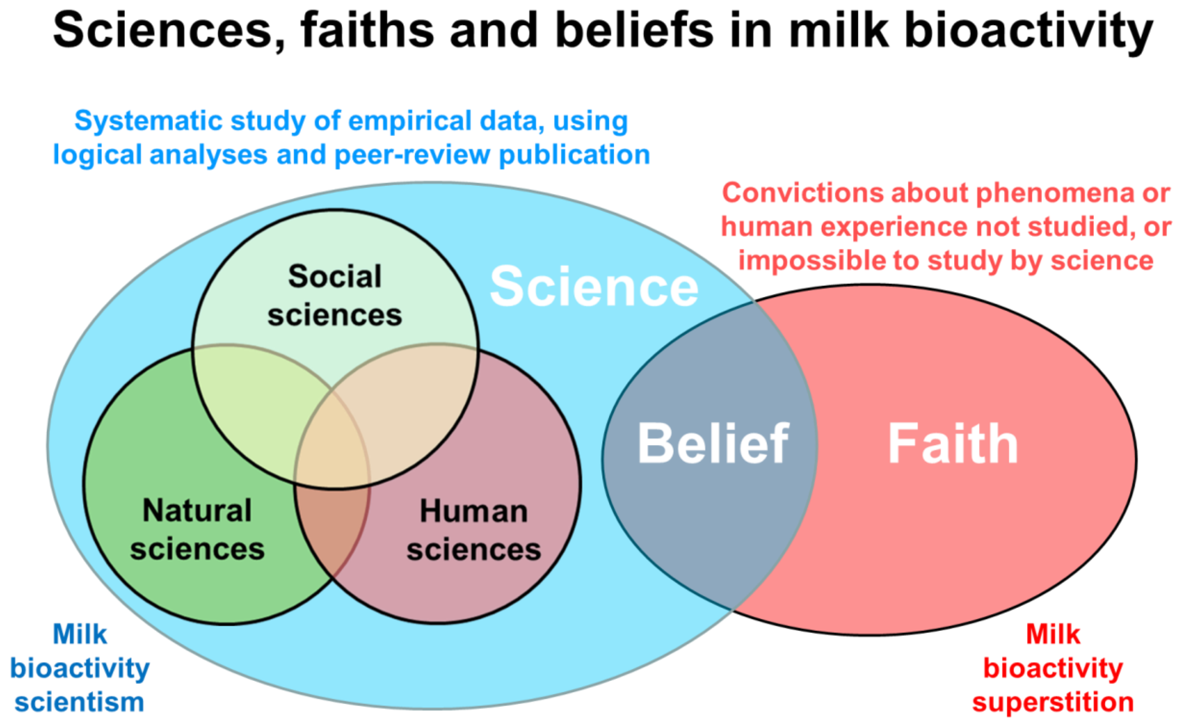


**Supplementary Figure 2.** Scientific information on milk bioactivity can be categorized as natural science, social science, or human science, depending on the research methodology and topic investigated. The vast majority of current milk bioactivity research is based on natural science, but insights from the other scientific domains are needed, especially when implementing milk bioactivity for human health. Beyond scientific evidence, 'faith' in various forms and expressions plays a role in the basic understanding and practical implementations of new dietary therapies. The concept of 'belief' represents an intermediate or combined position, relying partly on observation, data, and logic (science) but is combined with personal convictions, feelings, and intuitions not possible to verify by science (faith). See Figure 6 for the application of these themes to breastfeeding. 'Scientism' can be defined as trust in science to be the absolute and only justifiable access to true knowledge. Multiple sub-varieties of scientism exist [57]. Scientism reflects firm trust in the methodology and results of scientific studies as the only valid way to understand the natural and social world, including human health issues [195]. Traditionally, scientism refers mainly to natural science (structural/physical analyses from empirical data collection), not the qualitative analyses of society and human behavior in social or human sciences [57]. Some argue that modern medical scientism has serious negative consequences for advances in public health care [176]. The focus on natural science as the key source of genuine knowledge may lead to a highly materialistic and reductionist view of the complexity of human experience in relation to nature, society, and individuals. Scientism shares some similarity with religious faith and presumptions, especially the branches of redemptive or comprehensive scientism, holding views that science can and will eventually explain everything and solve all problems [57]. Scientism may, however, ignore the constant development of new questions, uncertainties, controversies, and reasonable doubts in scientific results and analyses, including in health sciences [108]. Scientism is common in relation to the practical use and implementation of milk bioactivity, e.g., the underlying assumption that all health effects of milk fractions or isolated milk bioactive proteins can be demonstrated by (natural) scientific analyses, if not now, then in the future. Milk bioactivity scientism is unjustified based on current scientific knowledge in relation to aspects of milk bioactivity that are yet unknown (see Figure 1). Results from (natural) science need to be coupled with other insights, ideas, and experience not easily proven by natural science [194], not even when coupled with insights from the social and human sciences. Some of these insights may reflect faith(s), e.g., deeply personal opinions, values, and meanings not possible to investigate or verify by any science. The term 'superstition' cannot be clearly distinguished from faith, but differs from faith in nature, origin, and perception by individuals and society [58,197]. It can be defined as 'beliefs or practices resulting from ignorance, fear of the unknown, trust in magic or chance, or a false conception of causation' (Merriam-Webster dictionary). Science, empirical evidence, and logical reasoning are important tools to combat such irrational superstition. Superstition often involves attributing certain events or

outcomes to specific actions, objects, or circumstances that are believed to have magical or mystical powers, even though there is no actual reason to assume causal connection. In contrast to beliefs (and to some degree faiths), superstition does not take advantage of science to understand the world [58]. Superstition often relies on anecdotal experience, folk traditions, and unfounded beliefs not based on any logical or historical foundation. Superstition lacks the depth of philosophical or spiritual understanding of faith, as embedded in organized religion or more generally in spirituality. While faith generates positive values, ethics, and behavior (e.g. compassion, empathy, and moral conduct), superstition leads to irrational behaviors or actions, often intended to avoid bad luck or seek good fortune. Superstition is considered as less legitimate than faith, even though both superstition and faith involve untestable convictions that go beyond empirical evidence. Accordingly, some ritualistic behaviors related to milk and its perceived health effects must be characterized as superstition, beyond rational belief or contested, spiritual faith.
